# In Situ Formation of Zwitterionic Ligands: Changing the Passivation Paradigms of CsPbBr_3_ Nanocrystals

**DOI:** 10.1021/acs.nanolett.2c00937

**Published:** 2022-05-24

**Authors:** Roberto Grisorio, Francesca Fasulo, Ana Belén Muñoz-García, Michele Pavone, Daniele Conelli, Elisabetta Fanizza, Marinella Striccoli, Ignazio Allegretta, Roberto Terzano, Nicola Margiotta, Paola Vivo, Gian Paolo Suranna

**Affiliations:** † Dipartimento di Ingegneria Civile, Ambientale, del Territorio, Edile e di Chimica (DICATECh), Politecnico di Bari, Via Orabona 4, 70125 Bari, Italy; ‡ CNR NANOTEC − Istituto di Nanotecnologia, Via Monteroni, 73100 Lecce, Italy; § Dipartimento di Scienze Chimiche, Università di Napoli Federico II, Complesso Universitario di Monte Sant’Angelo, Via Cintia 21, 80126 Napoli, Italy; ∥ Dipartimento di Fisica “Ettore Pancini”, Università di Napoli Federico II, Complesso Universitario di Monte Sant’Angelo, Via Cintia 21, 80126 Napoli, Italy; ⊥ Dipartimento di Chimica, Università degli Studi di Bari “A. Moro”, Via Orabona 4, 70126 Bari, Italy; # CNR−Istituto per i Processi Chimico Fisici, UOS Bari, Via Orabona 4, 70126 Bari, Italy; ∇ Dipartimento di Scienze del Suolo, della Pianta e degli Alimenti, Università degli Studi di Bari “Aldo Moro”, Via G. Amendola 165/A, 70126 Bari, Italy; ○ Hybrid Solar Cells, Faculty of Engineering and Natural Sciences, Tampere University, P.O. Box 541, FI-33014 Tampere, Finland

**Keywords:** perovskite nanocrystals, zwitterionic ligand, colloidal stability, DFT calculations, surface binding energy

## Abstract

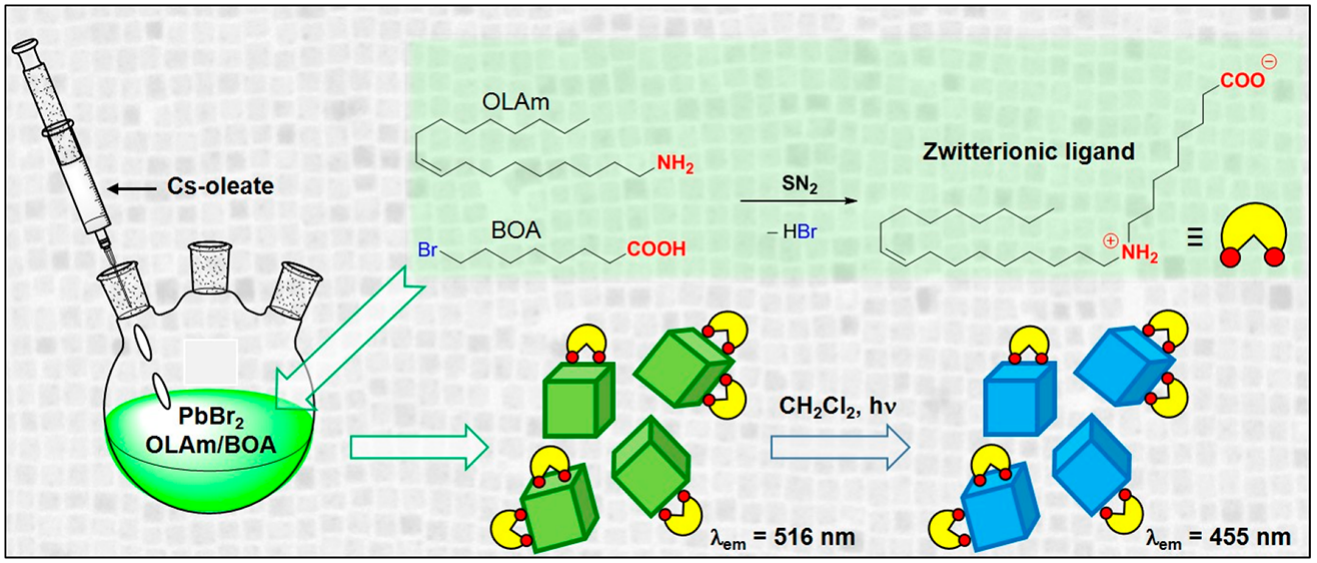

CsPbBr_3_ nanocrystals (NCs) passivated by conventional lipophilic capping ligands suffer from colloidal and optical instability under ambient conditions, commonly due to the surface rearrangements induced by the polar solvents used for the NC purification steps. To avoid onerous postsynthetic approaches, ascertained as the only viable stability-improvement strategy, the surface passivation paradigms of as-prepared CsPbBr_3_ NCs should be revisited. In this work, the addition of an extra halide source (8-bromooctanoic acid) to the typical CsPbBr_3_ synthesis precursors and surfactants leads to the *in situ* formation of a zwitterionic ligand already before cesium injection. As a result, CsPbBr_3_ NCs become insoluble in nonpolar hexane, with which they can be washed and purified, and form stable colloidal solutions in a relatively polar medium (dichloromethane), even when longly exposed to ambient conditions. The improved NC stability stems from the effective bidentate adsorption of the zwitterionic ligand on the perovskite surfaces, as supported by theoretical investigations. Furthermore, the bidentate functionalization of the zwitterionic ligand enables the obtainment of blue-emitting perovskite NCs with high PLQYs by UV-irradiation in dichloromethane, functioning as the photoinduced chlorine source.

Since the first report in 2015,^1^ cesium lead-halide perovskites nanocrystals (CsLHP NCs) are currently at the forefront of research on emissive materials with marked propensities for light-harvesting technologies and optoelectronic applications.^2−4^ However, the huge potential of CsLHP NCs is undermined by the ionic nature of the perovskite lattices, which induces highly dynamic bindings between the NC surface and its organic capping ligands. Typically, the passivating agents consist of ionic pairs of an anion (halide or carboxylate) and a cation (cesium or alkylammonium),^5^ resulting in a facile ligand cleavage from the NC surface, due to protonation/deprotonation of the carboxylate/alkylammonium couples favored by air and moisture exposure of the colloidal solutions.^6^ These processes manifest themselves with the loss of colloidal stability and structural integrity of the perovskite NCs,^7,8^ and the conventional purification approaches (based on their precipitation with polar solvents) dramatically amplify the effects of proton transfer processes and remove most of the capping ligands from the NC surface, compromising their structural integrity.^9^

Remarkable progress in obtaining stable CsLHP NCs has been reached by strengthening the binding of the capping ligands to the NC surface with robust passivating agents identified in quaternary dimethyldidodecylammonium bromide,^10−13^ zwitterionic compounds,^14−18^ alkylphosphonic acids,^19,20^ bidentate agents,^21,22^ and electron-donating ligands.^23−25^ Due to their poor availability and/or low solubility in the reaction conditions (particularly evident for ionic species),^26^ these capping ligands are often introduced onto the NC surface with onerous postsynthetic approaches. Although leading to an effective improvement of the optical properties and/or stability of the modified NCs, these postpreparative methods can also trigger irreversible structural, morphological, and spectroscopic transformations of the recipient NCs.^27^

To overcome these problems, in this work we have developed a straightforward synthetic procedure for passivating CsPbBr_3_ NCs through the *in situ* formation of a zwitterionic ligand via the SN_2_ reaction between an additional halide source (8-bromooctanoic acid) and oleylamine used as the surfactant. The formed zwitterionic ligand can adhere to the NC surface through the synergistic interaction with both the dialkylammonium and the carboxylate functionalities. This effective passivation reduces the solubility of the resulting NCs in nonpolar hexane, which can thus be used for the purification stages.

As shown in Figure 1A, along with the conventional PbBr_2_ precursor, the proposed synthetic protocol exploited an additional bromide source (8-bromooctanoic acid, BOA), which is endowed with a potential ligand functionality through the carboxylic group, providing “extra” halide anions (Supporting Information for details). The main reaction occurring during the incubation time (before cesium introduction) is schematically depicted in Figure 1B. The OLAm nucleophile can generate bromide ions by the SN_2_ reaction involving the only electrophile present in the reaction mixture (BOA, containing a bromine leaving group), yielding a bifunctionalized ligand, which prevalently exists in solution as a zwitterion containing the dialkylammonium and the carboxylate moieties (*vide infra*), both potentially interacting with the NC surface (Figure 1B). After the NC isolation following the removal of the supernatant solution from the first centrifugation, the precipitated fluorescent NCs resulted in being insoluble in hexane, allowing us to design a washing protocol without employing aggressive polar solvents (methyl acetate, methanol, or acetone). The purification of NCs was thus carried out by washing them twice with hexane, before the final dispersion in DCM, as described in Figure 1C.

**Figure 1:**
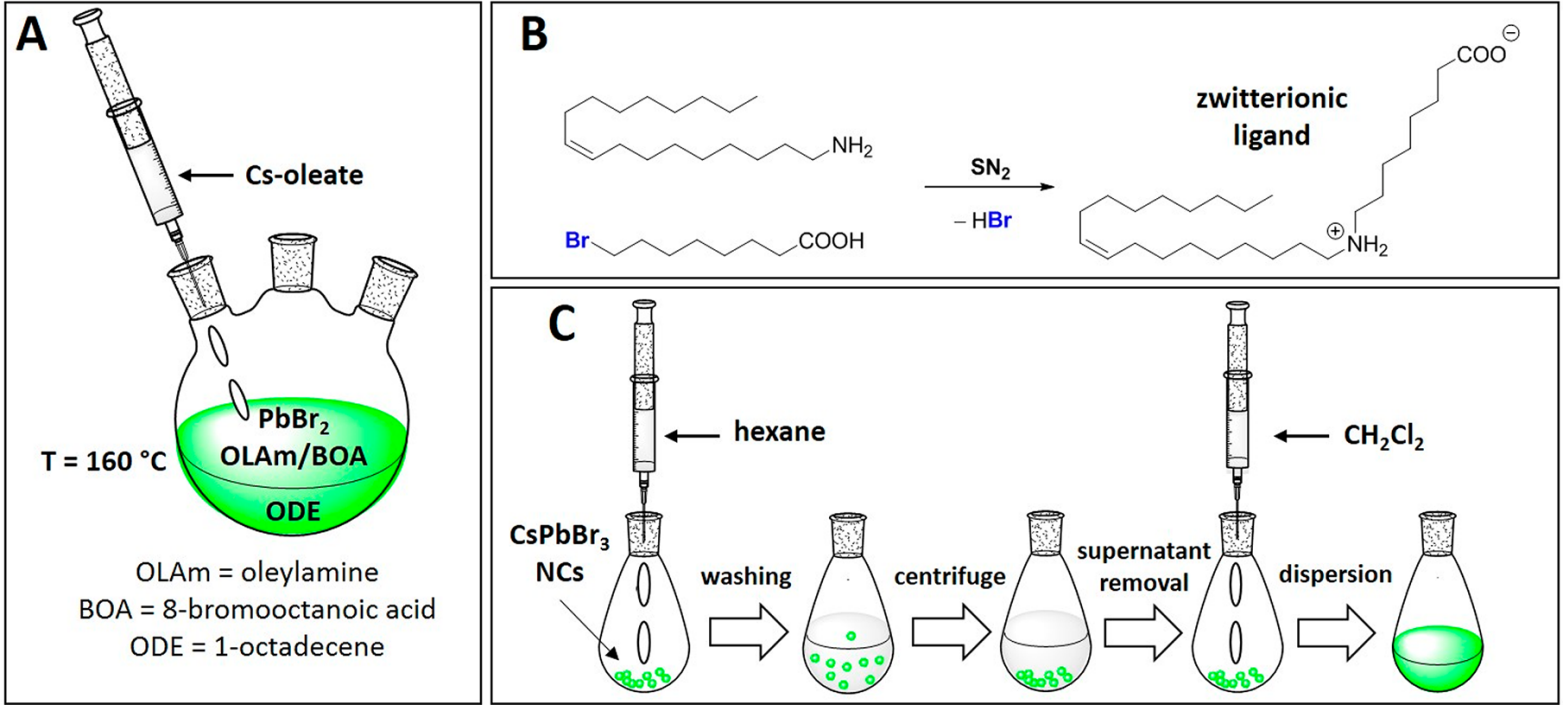
(A) Schematic representation of the synthetic approach for the obtainment of CsPbBr_3_ NCs. (B) The reaction involving the pristine surfactants and leading to the formation of the zwitterionic ligand, which occurs during the incubation stage before cesium introduction. (C) Schematization of the purification steps of the CsPbBr_3_ NCs upon removal of the supernatant from the first centrifugation, involving hexane as the washing solvent and DCM as the medium for storing the purified NCs dispersion.

To rationalize this unconventional behavior, we synthesized a batch of CsPbBr_3_ NCs under the same reaction conditions except for the use of 1-bromooctane (BO) as the additional bromide source in the substitution of BOA, therefore excluding the formation of the zwitterionic ligand (Scheme S2).^28^ As predictable, the obtained fluorescent material directly isolated from the reaction mixture after the centrifugation resulted in being colloidally dispersible in hexane. Therefore, the different behavior exhibited by CsPbBr_3_ NCs in terms of colloidal stability/instability in relation to the employed dispersing medium can only be ascribed to the presence of the zwitterionic ligand.

To ascertain the role of the zwitterion in the surface passivation, we investigated the surface chemistry of our bifunctional ligand-modified CsPbBr_3_ NCs by nuclear magnetic resonance (^1^H NMR) analyses. As shown in Figure 2A, the full ^1^H NMR spectrum of an aliquot of the reaction mixture produced before cesium injection and dissolved in CDCl_3_ was compared to the spectra of the pristine ligands (OLAm and BOA). The comparison evidenced the complete conversion of BOA during the incubation period, as confirmed by the disappearance of the proton signals attributable to its peculiar −C*H*_2_Br functionality (Figure 2A; Supporting Information for details). This observation implies the evolution of new organic species (not containing the −C*H*_2_Br functionality), which simultaneously generate bromide ions in the reaction mixture (Figures S1 and S2).

**Figure 2:**
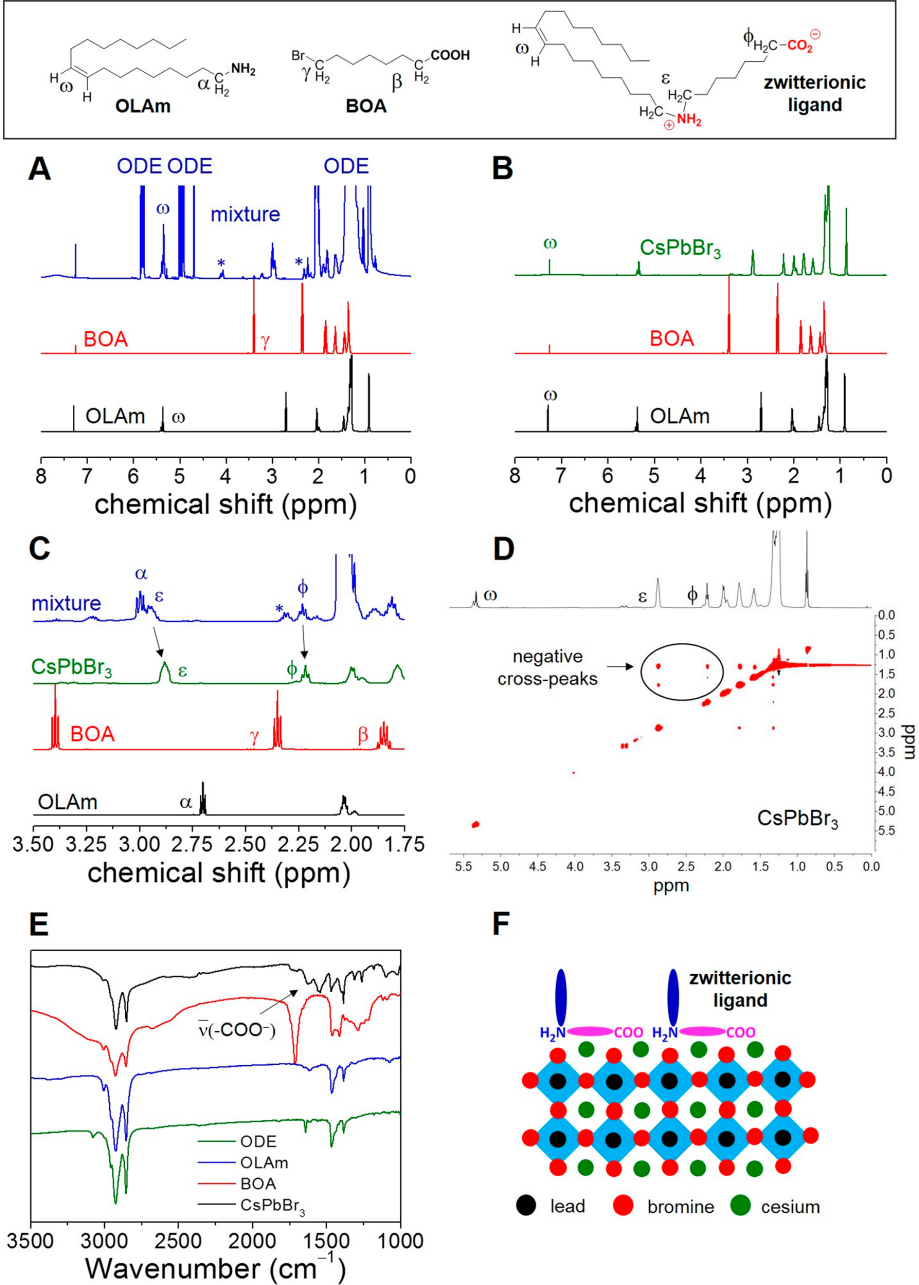
(A) Comparison between the full ^1^H NMR spectra (CDCl_3_) of oleylamine (OLAm), 8-bromooctanoic (BOA), and of the reaction mixture composed of PbBr_2_/OLAm/BOA (1/16/8 molar ratio) kept at 160 °C for 1 h in octadecene (ODE). The signals marked with an asterisk are attributable to the lactone byproduct (Scheme S1). (B) Comparison between the full ^1^H NMR spectra (CDCl_3_) of the purified CsPbBr_3_ NCs and of the pristine ligands. (C) Expansion of the ^1^H NMR spectral region containing the diagnostic signals of the pristine ligands (OLAm and BOA), of the reaction mixture after the incubation time, and of the purified CsPbBr_3_ NCs. (D) 2D-NOESY spectrum of the purified CsPbBr_3_ NCs recorded in CDCl_3_. (E) Comparison between the FT-IR spectra (KBr) of ODE, OLAm, BOA, and the purified CsPbBr_3_ NCs. (F) Schematization of the passivation mode (adsorption) at the NCs CsBr-rich surface involving the zwitterionic ligand formed during the incubation stage.

The ^1^H NMR spectrum of purified CsPbBr_3_ NCs clearly evidence a different composition of the organic shell with respect to the pristine ligands (Figure 2B). The most striking aspect is the absence of other contaminants, including the residual reaction solvent (ODE), and only two washing cycles with hexane are needed to remove all contaminant species weakly bound to the NC surface.

To reveal the nature of the organic species bound to the surface of our NCs and rationalize the peculiar behavior of their organic shell,^29^ we inspected the diagnostic ^1^H NMR region ascribable to the protons adjacent to the functional groups of the capping ligand (Figure 2C; Supporting Information for details). In the case of the purified CsPbBr_3_ NCs, only two sets of proton signals clearly attributable to the dialkylammonium (ε) and the carboxylate (ϕ) functionalities are observable in the ^1^H NMR spectrum, while the ratio between their integrals (ε:ϕ = 2:1) confirms that the bidentate ligand is the only structure composing the organic shell of our NCs. The interaction of the zwitterionic ligand with the NC surface was confirmed by 2D-NOESY investigations, which evidenced the generation of negative cross-peaks associable to proton signals of the bidentate passivating agent (Figure 2D). The interaction of the zwitterionic ligand with the NC surface through charged moieties was also ascertained by IR analyses, which showed the presence of a broad band (centered at ∼1540 cm^–1^) ascribable to the carboxylate functionality and absent in the spectra of the pristine ligands (Figure 2E).

We analyzed the elemental composition of the inorganic core of our NCs via scanning electron microscope coupled with energy dispersive X-ray spectroscopy (SEM-EDS). The experimentally observed elemental composition of our NCs (Cs_1.2_Pb_1.0_Br_3.1_) suggests a slight excess of cesium and bromine with respect to stoichiometry, indicating that a significant contribution to the NC passivation involving the zwitterionic ligand should occur through adsorption onto CsBr-rich surfaces (Figure 2F).

In order to shed light on the passivation mechanism and binding mode of the zwitterionic ligand, we carried out a DFT-based computational study on the CsBr-terminated (010) perovskite surface (Supporting Information for details). As shown in Figure 3, the bidentate mode (denoted as *COO+NH*_2_) is significantly more stable than the corresponding open configuration modes,^30^ in which binding occurs through one of the carboxylate oxygens (denoted as *COO*) or through the dialkylammonium group (denoted as *NH*_2_).^12,31,32^ This holds true for calculations performed both in vacuum and in solvents of different polarity (hexane or DCM). According to our calculations, monodentate binding with the dialkylammonium group is not stable at high surface coverages (Θ, 0.4 ligand/nm^2^) and relaxes to the *COO+NH*_2_ bidentate mode configuration. We obtained a strong binding energy (*E*_b_ = −3.81 eV) for the zwitterionic ligand in the bidentate binding mode (*COO+NH*_2_) to this perovskite surface in DCM at low Θ (0.1 ligand/nm^2^), which explains the remarkable stability of the purified CsPbBr_3_ NCs in such medium (*vide infra*). Binding energies (Figure S3) and short binding atom-surface distances (Figure S4–S5) suggest strong adsorption of the bidentate zwitterionic ligand to the ideal CsPbBr_3_ surface, which accounts for the removal of other possible passivating agents during the washing stages of the NCs with hexane.

**Figure 3:**
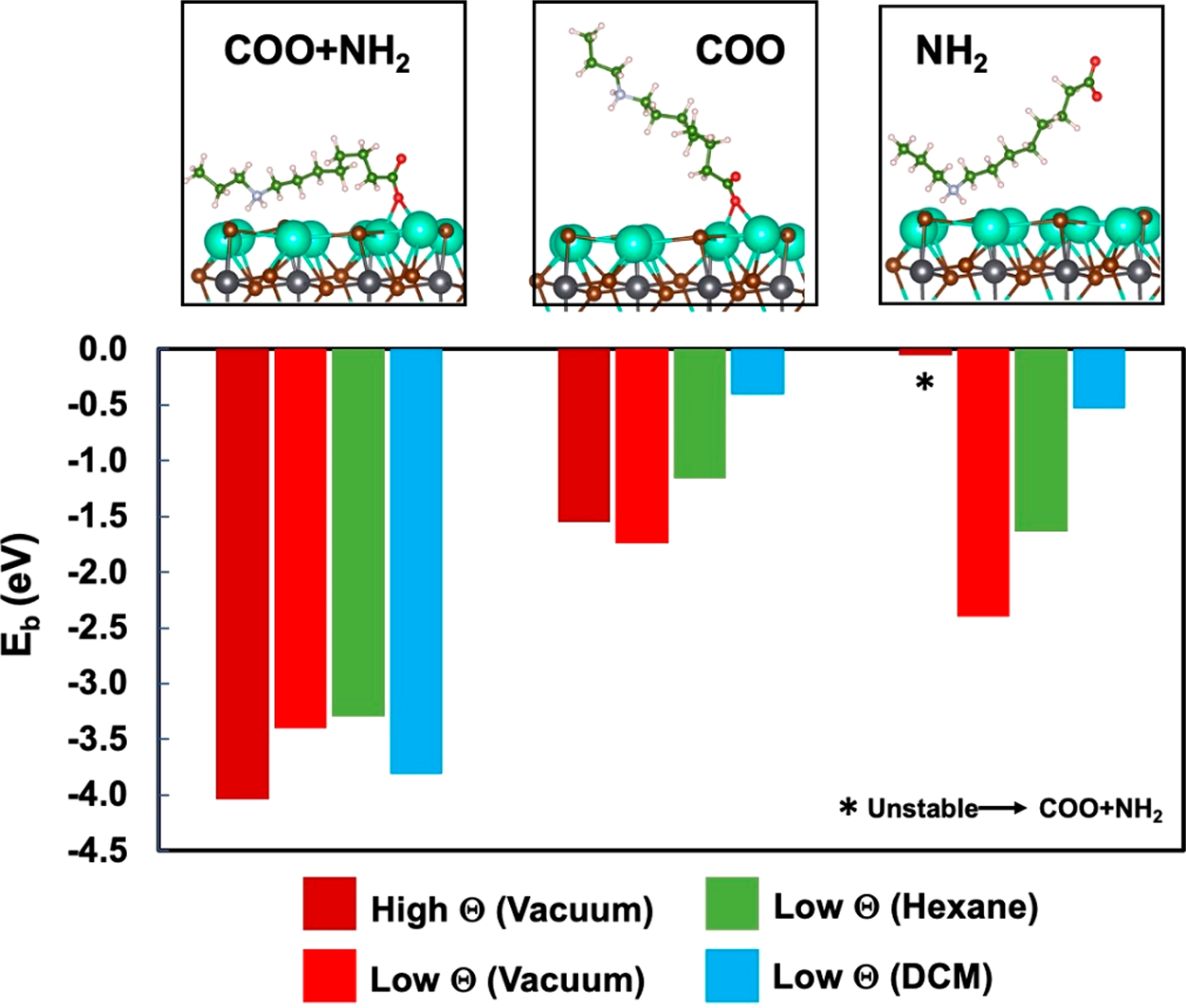
Lateral views and computed binding energies (*E*_b_) for the zwitterionic ligand on the CsPbBr_3_ (010) stoichiometric surface at different coverages (Θ) and dielectric media featuring both carboxylate and dialkylammonium (*COO+NH*_2_), carboxylate (*COO*) or dialkylammonium (*NH*_2_) as anchoring groups. Atom color labels: C (green), H (light pink), O (red), N (light blue), Cs (turquoise), Pb (gray), and Br (brown).

We ascertained that also the presence of surface point defects favors the bidentate anchoring of the zwitterionic ligand (*Def-COO+NH*_2_ mode in Figure 4 and Figures S6–S7), as this configuration is stable both in DCM (*E*_b_ = −5.37 eV) and in hexane (*E*_b_ = −4.44 eV). Furthermore, a different bidentate binding mode, denoted as *Def-COO+NH*_2_*(CH*_2_*)* in Figure 4, resulted in an efficient passivation of the NC defective surface (*E*_b_ = −2.31 to −3.23 eV). In such a configuration, the Cs^+^ vacancy is filled by a methylene group of the aliphatic chain, while the dialkylammonium group forms hydrogen bonds with the adjacent bromine atoms.

**Figure 4:**
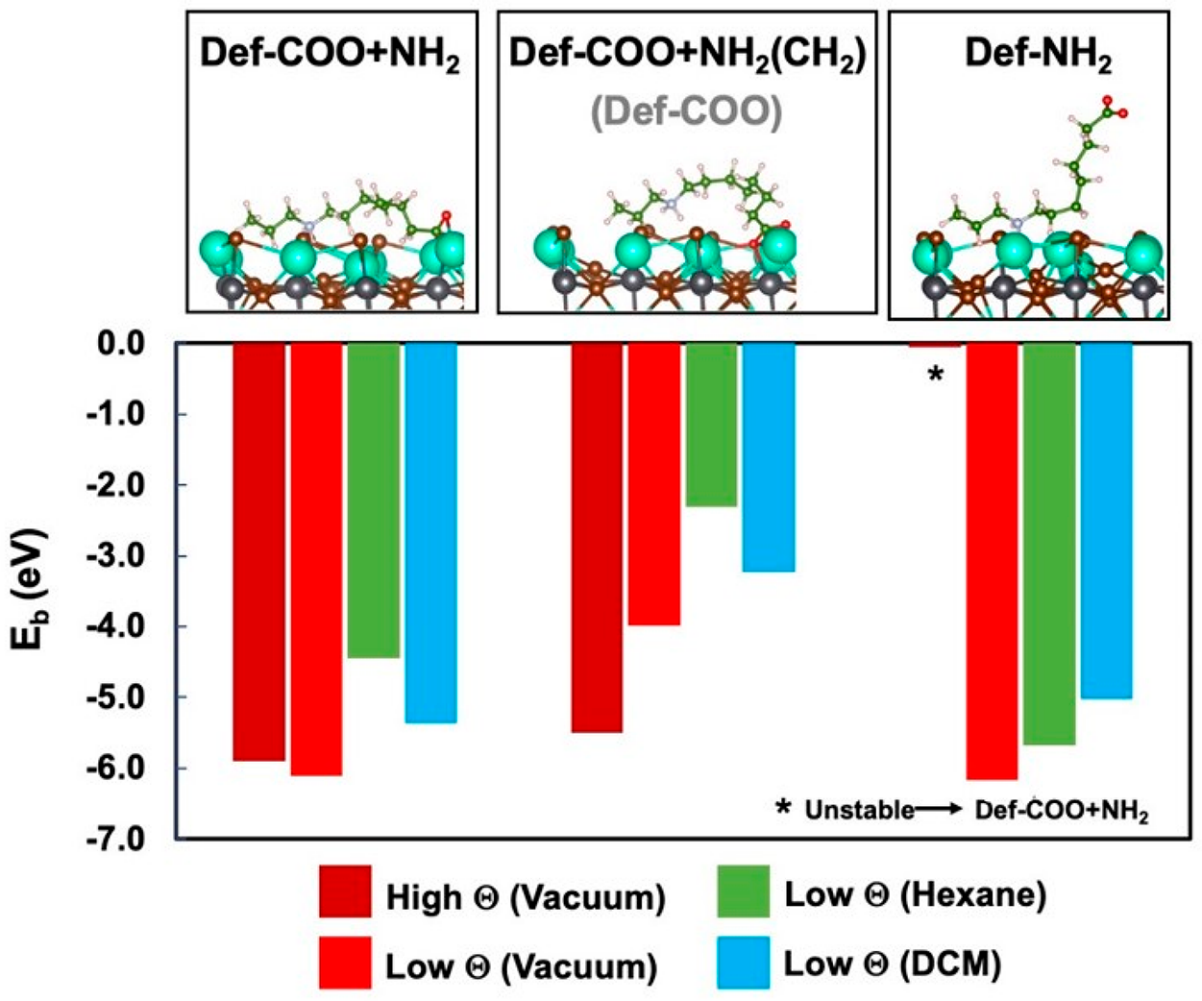
Lateral views and computed binding energies (*E*_b_) for the zwitterionic ligand on CsPbBr_3_ (010) defective surface containing one Cs^+^ and one Br^–^ vacancies at different coverages (Θ) and dielectric media. The bidentate (*Def-COO+NH*_2_) and the corresponding monodentate (*Def-COO* or *Def-NH*_2_) anchoring modes are computed. The *Def-COO* anchoring mode is not stable and evolves toward a *Def-COO+NH*_2_*(CH*_2_*)* bidentate binding mode. Atom color labels: C (green), H (light pink), O (red), N (light blue), Cs (turquoise), Pb (gray), and Br (brown).

Conversely, the monodentate binding mode (*Def-NH*_2_) is stable only at low surface coverages and evolves to the expected bidentate mode with the dialkylammonium and the carboxylate moieties saturating Cs^+^ and Br^–^ vacancies, respectively, for high surface coverages. It is important to note that in the *Def-NH*_2_ binding mode at low surface coverages, which is remarkably stable in hexane (*E*_b_ = −5.67 eV), the zwitterionic ligand provides a polar surrounding to the NC surface due to the peripheral carboxylate group, and could be held responsible for the NC insolubility in hexane.

The morphological assessment of the synthesized NCs, performed by transmission electron microscopy (TEM), revealed the formation of nanocubes with average sizes of 21.4 ± 4.1 nm (Figure S8). The formation of relatively large nanoparticles can be ascribed to the presence of dialkylammonium-based ligands, which do not efficiently compete with cesium cations during crystal growth. The optical characterization of purified CsPbBr_3_ NCs revealed a sharp excitonic absorption peak at 505 nm and a narrow PL emission band centered at 516 nm (fwhm = 18 nm, Figure 5A). The relatively small Stokes shift suggests that the emission photons exclusively emerge from direct exciton recombination. Analogously to the nanoparticles with a low surface coverage,^33^ the emission intensity of our purified CsPbBr_3_ NCs measured in diluted DCM solution (PLQY = 89%) is comparable with that of the as-synthesized CsPbBr_3_ NCs without the zwitterionic passivating agents (PLQY = 95%, Figure S9). In order to gain insight into the exciton recombination dynamics, we carried out time-resolved PL measurements (Figure 5B), which have evidenced a single lifetime component (4.0 ns) associated with the high QY value, thus confirming the excellent optical properties of our purified CsPbBr_3_ NCs.

**Figure 5:**
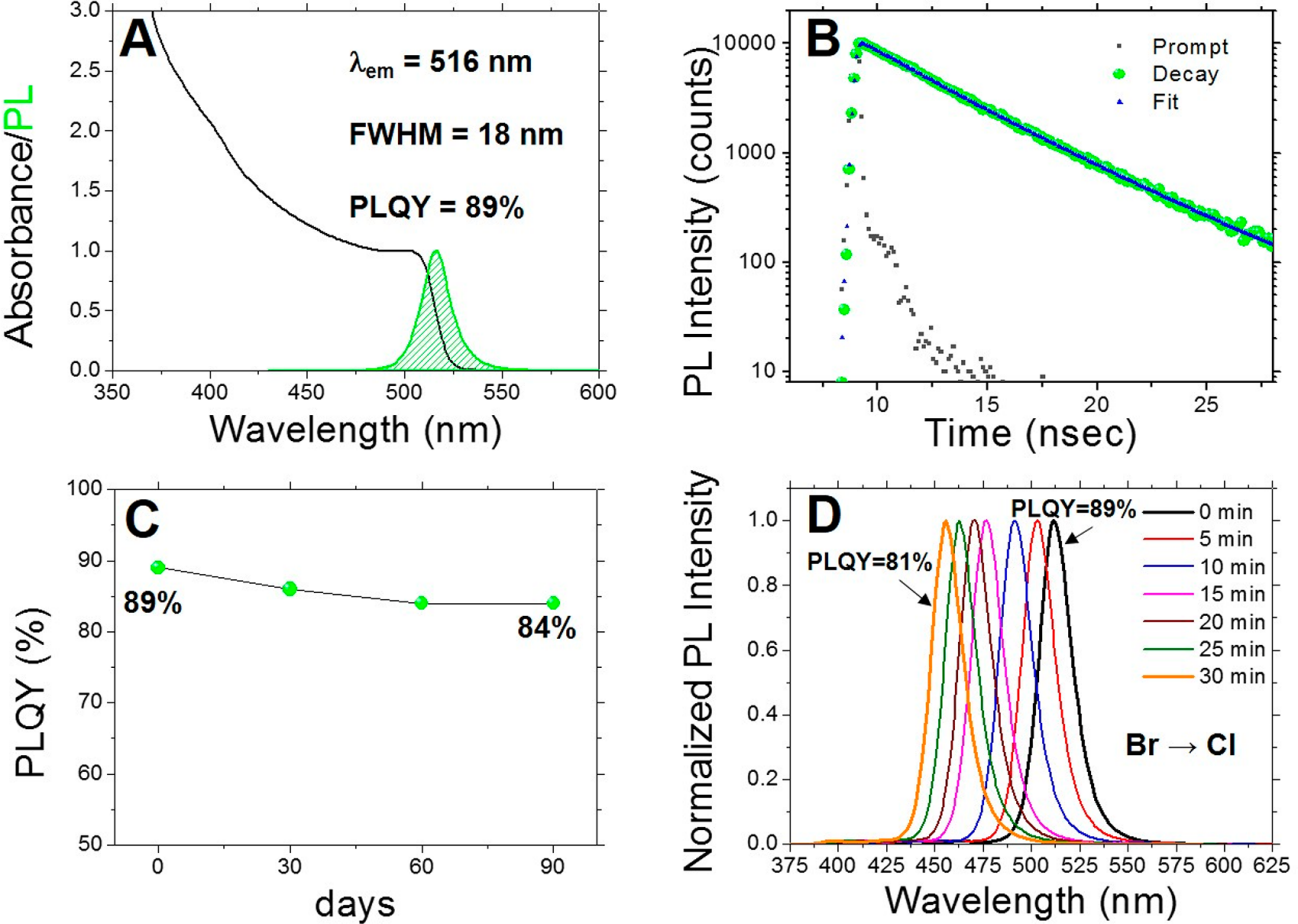
(A) UV–vis and PL spectra of CsPbBr_3_ NCs recorded in DCM. (B) Time-resolved photoluminescence decay of CsPbBr_3_ NCs recorded in DCM. (C) Evolution of the PLQY of the purified CsPbBr_3_ NCs stored in DCM under ambient conditions. (D) Evolution of the PL profiles of the purified CsPbBr_3_ NCs under UV-irradiation (365 nm, 8 W/cm^2^) in DCM solution.

The compatibility of DCM with the long-term stability of the purified NCs was evaluated by monitoring the PLQY of their stock solution stored in ambient conditions. As shown in Figure 5C, the PLQY of our CsPbBr_3_ NCs did not exhibit substantial variations over time (90 days), testifying the robustness of the passivation offered by the zwitterionic ligand. In comparison, the PLQY of as-synthesized conventional CsPbBr_3_ NCs stored in DCM was drastically degraded, dropping to values as low as 25% (Figure S9) during the same time. However, the main drawback concerning the use of DCM as the solvent for dispersing CsLHP NCs resides in the well-documented attitude of dihalomethanes to be photoreduced by perovskite NCs forming halide anions potentially available for halide-exchange processes.^34^ Remarkably, under indirect daylight, the emission spectra of CsPbBr_3_ NCs and the relevant PLQY remained unchanged over time when dispersed in DCM, indicating that no anion exchange occurs in the solution of NCs in their respective solvents used for their storage.

Since only the interfacial electron transfer from the photoexcited CsLHP NCs can promote the reductive dissociation of dihalomethanes,^35^ we exposed our NCs dispersed in DCM to UV-irradiation (365 nm, 8 W/cm^2^). In this case, we observed the progressive blue shift of their PL maxima upon UV irradiation (λ_em_ = 455 nm after 30 min of irradiation, Figure 5D), while the high PLQY (81%) exhibited by the generated mixed-halide CsLHP NCs at the end of the photoinduced process suggests that surface halide vacancies are not generated during the halide exchange. This result validates the beneficial role of the engineered organic shell in the photostability of our NCs under harsh irradiation conditions. In fact, the same photoinduced halogen exchange carried out on the CsLHP NC passivated by conventional capping ligands^34^ caused an analogous blue shift of the emission maxima (λ_em_ = 464 nm upon 30 min of irradiation) accompanied, however, by a remarkable drop of the corresponding PLQY (down to 26% after 30 min of irradiation), as shown in Figures S10–S11.

We also tested our synthetic approach on the preparation of iodine-based NCs by introducing PbI_2_ as the halide precursor in the same reaction conditions utilized in this work. For the obtained CsPbBr_*x*_I_3–*x*_ NCs, we verified a similar behavior with respect to that observed for CsPbBr_3_ NCs (Figures S12–S14).

In conclusion, the suitable engineering of the organic shell composition of CsPbBr_3_ NCs can reduce their affinity toward apolar solvents (nonaggressive toward the ionic structure of perovskites) allowing the use of hexane for the purification stages in alternative to conventional polar solvents. The CsPbBr_3_ NCs prepared following our protocol can be deprived of the organic contaminants without *de facto* compromise of their optical properties during the purification stages. The purified NCs are dispersible in DCM, in which they are found to be colloidally and optically stable for three months. Highly efficient blue-emitting NCs are obtained by UV-irradiation of the purified CsPbBr_3_ NCs passivated by the bidentate capping ligand in DCM as the solvent providing the chloride source for the photoinduced halide exchange.

## Supporting Information Available

Experimental details; additional spectroscopical investigations on conventionally prepared perovskite NCs; details on theoretical calculations. (PDF)
