# Supplementary material for: In Situ Formation of Zwitterionic Ligands: Changing the Passivation Paradigms of CsPbBr3 Nanocrystals
Source: Nano Lett. 2022 May 24;22(11):4437–44. doi: 10.1021/acs.nanolett.2c00937 (PMC9185741; doi:10.1021/acs.nanolett.2c00937)
Supplement: Supplementary file 1 — nl2c00937_si_001.pdf [file nl2c00937_si_001.pdf]

In-Situ Formation of Zwitterionic Ligands: Changing the Passivation Paradigms of $CsPbBr_3$ Nanocrystals

Roberto Grisorio*, Francesca Fasulo, Ana Belén Muñoz-García, Michele Pavone, Daniele Conelli, Elisabetta Fanizza, Marinella Striccoli, Ignazio Allegretta, Roberto Terzano, Nicola Margiotta, Paola Vivo and Gian Paolo Suranna

Chemicals. Cesium carbonate (Cs_2CO_3, 99.9% metals basis, Alfa Aesar), lead bromide ($PbBr_2$, 99.999% metals basis, Aldrich), 8-bromooctanoic acid (BOA, 97%, Aldrich), 1-bromooctane (99%, Aldrich), oleylamine (OLAm, technical grade 70%, Aldrich), oleic acid (technical grade 90%, Aldrich), 1-octadecene (ODE, technical grade 90%, Aldrich), hexane (Aldrich, HPLC grade), dichloromethane (Aldrich, HPLC grade).

Synthesis of $CsPbBr_3$ NCs capped by zwitterionic ligands. The syntheses were carried out under nitrogen atmosphere using standard Schlenk techniques. The cesium-oleate solution was prepared as follows: Cs_2CO_3 (0.407 g, 1.25 mmol), OLA (1.55 mL, 4.91 mmol), and ODE (20 mL) were mixed in a 50 mL Schlenk tube and kept in vacuum for 1 hour at 120 °C. After the complete solubilization of the salt, the tube was filled with nitrogen and kept at 120 °C before the injection. ODE (5.0 mL), lead bromide (0.073 g, 0.20 mmol), oleylamine (0.55 mL, 1.60 mmol) and 8-bromooctanoic acid (0.178 g, 0.80 mmol) were added to a 50 mL Schlenk tube and the obtained mixture was heated at 160 °C. After 1 h, the hot Cs-oleate solution (0.40 mL) was injected and the resulting colored mixture was rapidly cooled in an ice bath. Methyl acetate (8.0 mL) was added to the cooled reaction crude and the resulting suspension was centrifuged at 4000 rpm for 40 min. The supernatant solution was discarded, and the obtained precipitate was redispersed in DCM (2.0 mL). To obtain purified samples, hexane (2.0 mL) was added to the precipitate of the first centrifuge, the resulting suspension was centrifuged at 4000 rpm for 10 min and the supernatant was removed to collect the precipitate. Eventually, this operation can be repeated before the final redispersion in DCM (2.0 mL).

Conventional synthesis of $CsPbBr_3$ NCs. ODE (5.0 mL), lead bromide (0.063 g, 0.20 mmol), oleylamine (0.55 mL, 1.60 mmol), and 1-bromooctane (0.154 g, 0.80 mmol) were added to a 50 mL Schlenk tube and the obtained mixture was heated at 160 °C. After 1 h, the hot Cs-oleate solution (0.40 mL) was injected and the resulting colored mixture was rapidly cooled in an ice bath. After

centrifugation at 4000 rpm for 40 min, the supernatant solution was discarded, and the obtained precipitate was redispersed in hexane (2.0 mL).

Spectroscopical characterization. UV–vis absorption spectra were collected using a Jasco V670 spectrometer operating in transmission mode. Steady-state photoluminescence (PL) spectra from solutions were acquired on a Varian Cary Eclipse instrument. The quantum yields were determined by using fluorescein as standard, according to reported procedures.[1] FT-IR measurements were recorded on a JASCO 4200 spectrophotometer in attenuated total reflectance (ATR) mode.

Time-resolved photoluminescence (TRPL). The lifetime measurements were performed by Time-Correlated Single Photon Counting (TCSPC) by means a FluoroHub HORIBA Jobin-Yvon module. The pulsed excitation source was a laser diode emitting at 375 nm (NanoLED N375L) with pulse width < 200 ps, average power of 14pJ/pulse and a repetition rate of 1 MHz. The PL emission was detected by a picosecond photon counter TBX ps Photon Detection Module (HORIBA Jobin-Yvon)

Transmission electron microscopy (TEM) characterization. TEM micrographs were acquired using a JEOL JEM1011 microscope, operating at an accelerating voltage of 100 kV. The instrument was equipped with a tungsten electron source and a high-resolution CCD camera. Samples for TEM analysis were prepared by dipping a carbon-coated copper grid into the NC solution diluted with anhydrous *n*-hexane. Statistical size analysis (NC average size and standard deviation) was performed by using a freeware image analysis software (AxioVision); each sample contained not less than one hundred nanoparticles.

Nuclear Magnetic Resonance. ^{1}H-NMR spectra were recorded on an Agilent 500 MHz instrument. All chemical shifts were referred to the non-deuterated chloroform residue signal at 7.26 ppm. The NOESY spectra were acquired using standard pulse sequences; mixing time was set to 300 ms.

Field emission gun scanning electron microscopy coupled with energy dispersive X-ray spectroscopy. FEG-SEM-EDX analysis were performed using a Zeiss Σigma 300VP electron microscope equipped with an Oxford C-MaxN SDD detector with an active area of 20 mm^2. Perovskite NCs were suspended in DCM and deposited on aluminum stubs. The analysis was performed using a working distance of 7.5 mm, an acceleration voltage of 15 kV and a magnification of 1000×. The accuracy of the analysis was checked using the MAC (Micro-Analysis Consultants Ltd) reference materials.

Computational Details. We performed density functional theory (DFT)[2] calculations using the Perdew-Burke-Erzenhof (PBE)[3] exchange correlation functional, including Tkatchenko–Scheffler

(TS) correction[4,5] accounting for van der Waals dispersion forces, as implemented in the Fritz Haber Institute ab initio molecular simulations (FHI-aims) code.[6] In the FHI-aims framework, we employed the light–tier1 basis set of numerical atom-centered orbitals (NAO) for each atom.[7] The electrons were described by the zero–order regular approximation (atomic ZORA).[8] For the self-consistency of the electron density, we employed a total energy criterion of 1×10^{-6} eV. For calculations with periodic boundary conditions (PBC), we apply the surface-slab approach[9] to build up the structural models for $CsBrPb_3$ (010) surfaces by cleaving the CsBr termination from the bulk structure with optimized lattice constants (Figure S15c) and then introducing 25Å of vacuum along the c direction. Two supercells with different surface area of the xy plane have been considered in order to evaluate the adsorption behavior and energetics of the molecules at different coverage (Θ) levels (Figure S15d-e). The smallest supercell, i.e. the highest Θ, with neighboring image zwitterionic molecules ~ 13 Å/~ 5 Å apart in the xy plane, is a 2×2×5 layers of the (010) unit cell. The largest system is a 4×4×4 layers supercell with a low Θ of molecules separated by ~ 29 Å/~ 20 Å. (2×2×1) and gamma point (1×1×1) k-point sampling schemes are applied to the small and large supercells, respectively. During geometry optimizations involving the non-defective $CsPbBr_3$ surface, atoms of the bottommost layers have been fixed to their bulk-like positions, while the first layer and the adsorbed molecule have been allowed to relax. Defective $CsPbBr_3$ surfaces have been generated by removing one Cs and one Br atoms from the first exposed layer. In this case, both surface and subsurface layers have been relaxed together with the molecule during geometry optimizations. Our relaxed structures present maximum forces acting on each atom below 0.05 eV/Å. Geometry optimizations have been performed on both supercell sizes but, since adsorption structural features are virtually identical for both cells, we use the relaxed structures obtained in the smallest cell for single point calculations in the larger cell and for cluster calculations. Adsorption modes that present significant differences for high Θ and low Θ are discussed in detail in the main text. Effects of different solvents on the stability of the different anchoring modes have been studied with the efficient implicit solvation model (MPE)[10] in non-periodic calculations using a large cluster containing atoms from a 3.5×2.5×4 layers supercell rotated 45° (Figure S15f). Besides performing cluster calculations in vacuum to be compared with the PBC, low Θ one, we considered the DCM and hexane solvents with relative permittivity of 8.93 and 1.88, respectively. In high coverage, the supercell (16.47 Å × 16.58 Å) has an area of 2.73 nm^2; therefore, the coverage is 1 ligand / 2.73 nm^2 = 0.4 ligand/nm^2. In low coverage, the supercell area is four times bigger than the previous one and the coverage is 1 ligand / 10.92 nm^2 = 0.1 ligand/nm^2. The calculated binding energies (E_b) concerning the different anchoring modes of the zwitterionic ligand with the $CsPbBr_3$ NC surfaces were modeled by maintaining the original chain separation (C8) between the

dialkylammonium and the carboxylate groups, while introducing a shorter group (–C_3H_8) to take into account the oleyl fragment.

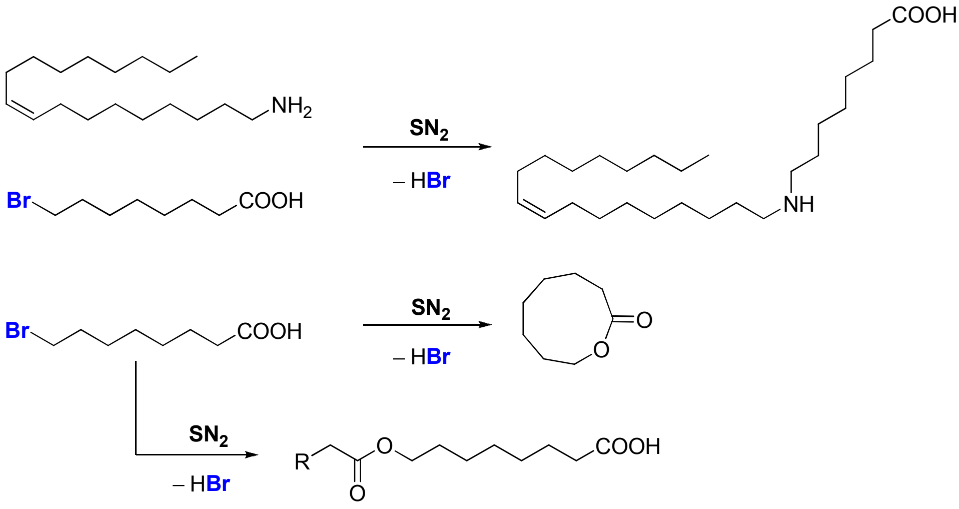

Scheme S1. Overview of all possible reactions potentially occurring during the incubation stage. A mixture composed of lead bromide, oleylamine (OLAm), and BOA in 1-octadecene (ODE) was heated at the chosen reaction temperature (160 °C) for 1 h before the introduction of the Cs-oleate solution. The $CsPbBr_3$ NCs in the presence of the zwitterionic capping ligand were obtained by setting the $PbBr_2$:BOA molar ratio at 1:8, while the molar ratio of the other ligand (OLAm) with respect to BOA was fixed at 2:1. The intermolecular condensation of BOA, leading to the formation of acyclic esters, should be ruled out as suggested by the absence of residual $–CH_2Br$ signals in the relevant ^1H-NMR spectrum (Figure 2 of the main text). The formation of the secondary amine rather than the ester is probably favored by the excess of OLAm employed. The other possible products of the incubation reaction involve the intra- or inter-molecular reactivity of BOA forming the corresponding lactone or longer-chain carboxylic acids, respectively. The twofold stoichiometric excess of OLAm with respect to BOA was thus imposed to sequestrate the hydrobromic acid byproduct of the S_N2 reaction in the mixture, promoting the formation of the zwitterionic ligand.

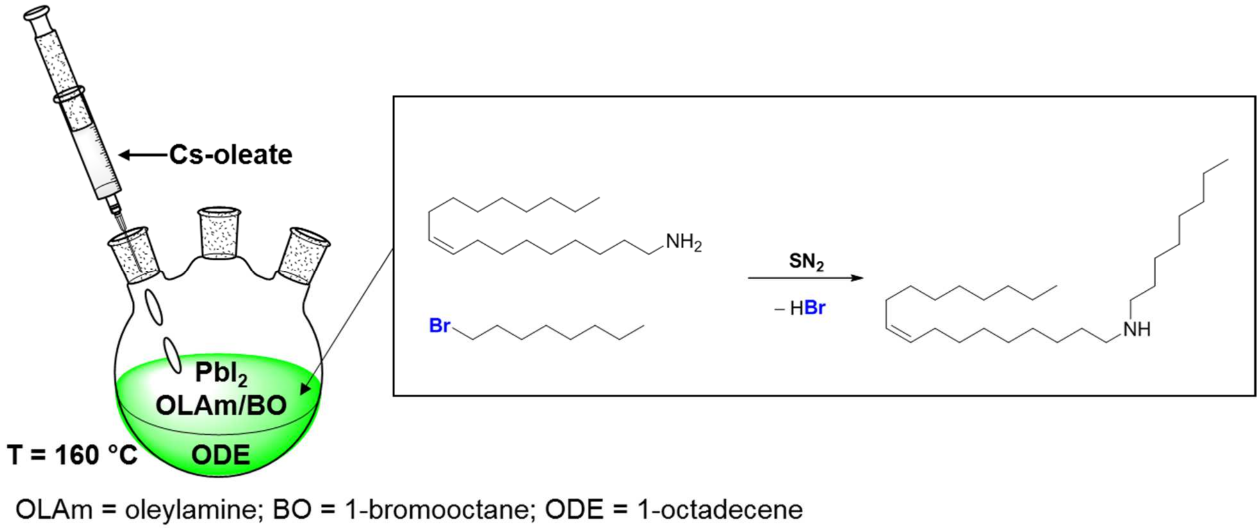

Scheme S2. Schematic representation of the "conventional" synthesis of $CsPbBr_3$ NCs ($PbBr_2$:BO = 1:4), where the bromide anions evolve by the SN_2 reaction between oleylamine and 1-bromooctane. We stress that the purification stage with polar antisolvents drastically degrades the PLQY (to 53%) of the $CsPbBr_3$ NCs prepared by conventional synthetic approaches.

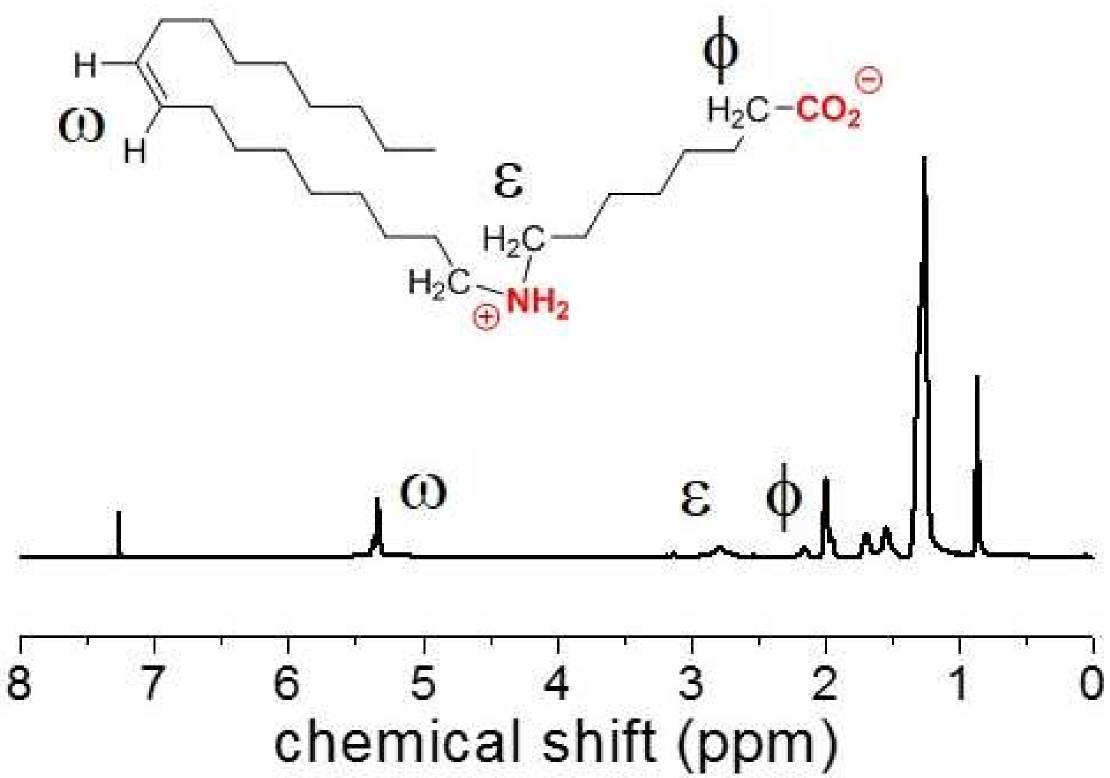

Figure S1. ^{1}H-NMR spectrum ($CDCl_3$) of the zwitterionic ligand formed by reacting OLAm and BOA (2:1 mol:mol) in triethylamine at 60 °C for 24 h. After the reaction, the formed white precipitate was filtered and washed with hexane. The obtained crude product was then dissolved in $CHCl_3$ and washed with water (3×). Noteworthy, the chemical shifts of the ^{1}H-NMR signals (apart from the broadness of the peaks ascribable to ε and ϕ protons) observed for this species are superimposable with those ascribed to the zwitterionic ligand formed in the reaction mixture and passivating the $CsPbBr_3$ NCs (Figure 2 of the manuscript).

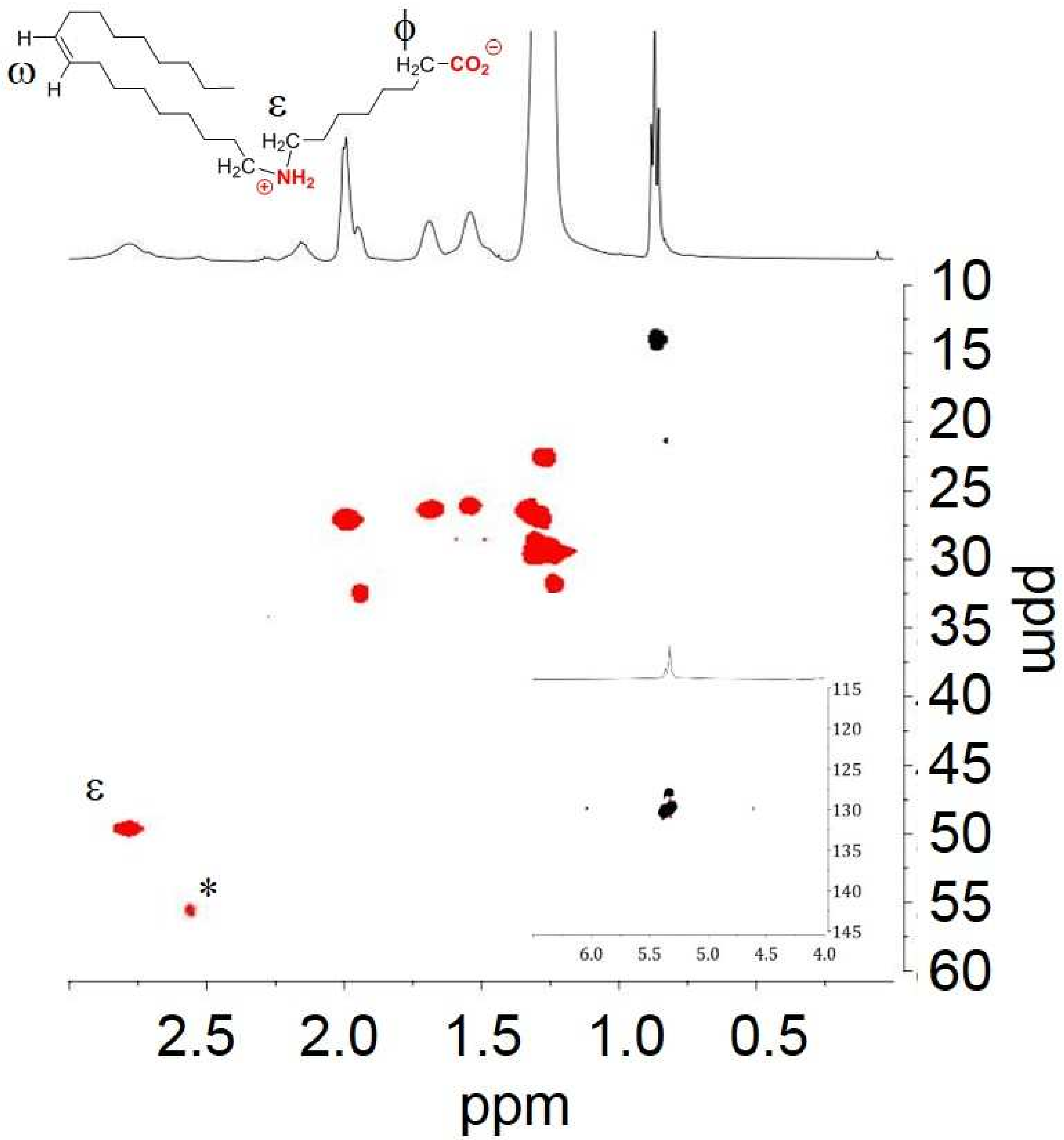

Figure S2. ^{1}H-^{13}C HSQC ($CDCl_3$) of the zwitterionic ligand prepared in triethylamine. Cross peaks with opposite phases are shown in red for CH_2 and in black for CH/CH_3. The cross-peak corresponding to the ϕ protons was not observed, probably due to the proton exchanges typical of the free zwitterionic ligands. (Inset) The double bond region. (*) Byproduct.

Appendix to the NMR discussion: Through an inspection of the chemical shift of the ^{1}H-NMR signals evolved during the incubation time, the formation of new organic species can effectively be deduced. In detail, the signal patterns are compatible with the formation of the zwitterionic ligand resulting from the SN_2 reaction between OLAm and BOA (Figure S1 and S2), and of the lactone deriving from the intramolecular cyclization of BOA (Scheme S1). The peculiar ^{1}H-NMR signals ascribable to the lactone (devoid of functional groups for surface passivation) are marked with an asterisk in the corresponding spectrum of the reaction mixture (Figure 2A). During the incubation time, the generation of the zwitterionic ligand is evident through its diagnostic proton signals (marked with ε and ϕ in the corresponding ^{1}H-NMR spectrum) along with the residual presence of OLAm (as the corresponding conjugated acid), which is characterized by the –CH_2NH_2 signal marked with α (Figure 2C).

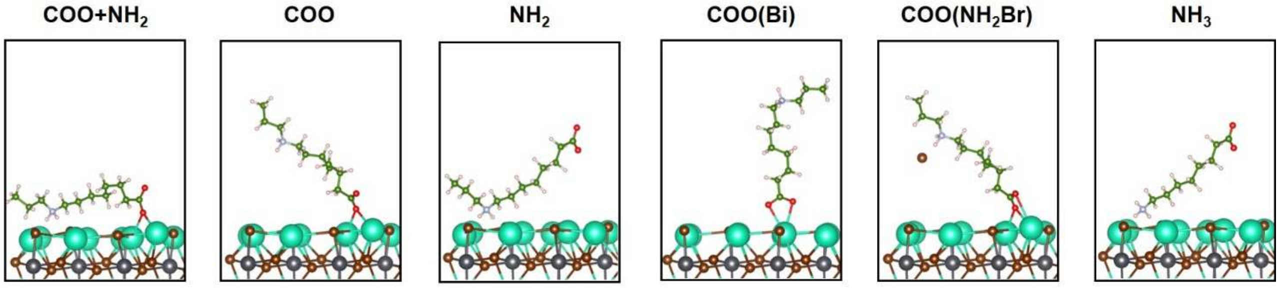

$$E_b = E_{Surf\text{-}Mol} - E_{Surf} - E_{Mol}$$

E_b (eV)	COO-NH_2	COO	NH_2	COO(Bi)	COO(NH_2Br)	NH_3
High Θ<br>2x2 Supercell, Vacuum	-4.04	-1.55	**Unstable**	-0.61	-1.48	-1.10
Low Θ<br>4x4 Supercell, Vacuum	-2.94	-1.43	-2.87	-2.25	-0.31	-1.31
Low Θ<br>Cluster, Vacuum	-3.40	-1.74	-2.40	-1.24	-2.40	-1.73
Low Θ<br>Cluster, Hexane	-3.30	-1.16	-1.64	-0.86	-1.64	-1.97
Low Θ<br>Cluster, DCM	-3.81	-0.41	-0.53	-0.32	-0.53	-0.49

Figure S3. Computed binding energies (E_b) for all anchoring configurations of the zwitterionic molecule considered in this work on $CsPbBr_3$ stoichiometric surface at different coverages (Θ) and dielectric media. Data highlighted in yellow are featured in Figure 3 of the main text. Color legend: C (green), H (light pink), O (red), N (light blue), Cs (turquoise), Pb (grey) and Br (brown). The configuration in which the zwitterionic molecule is perpendicularly arranged with respect to the NC surface and bound through the two oxygens of the carboxylate group to the cesium atom (a chelating mode) is denoted as *COO(Bi)*. We deemed necessary, for comparison, to investigate the anchoring mode (denoted as *NH_3*) of a suitable alkylammonium ligand modelling the OLAm surfactant, in which the peripherical carboxylate group was introduced to formally preserve the charge neutrality.

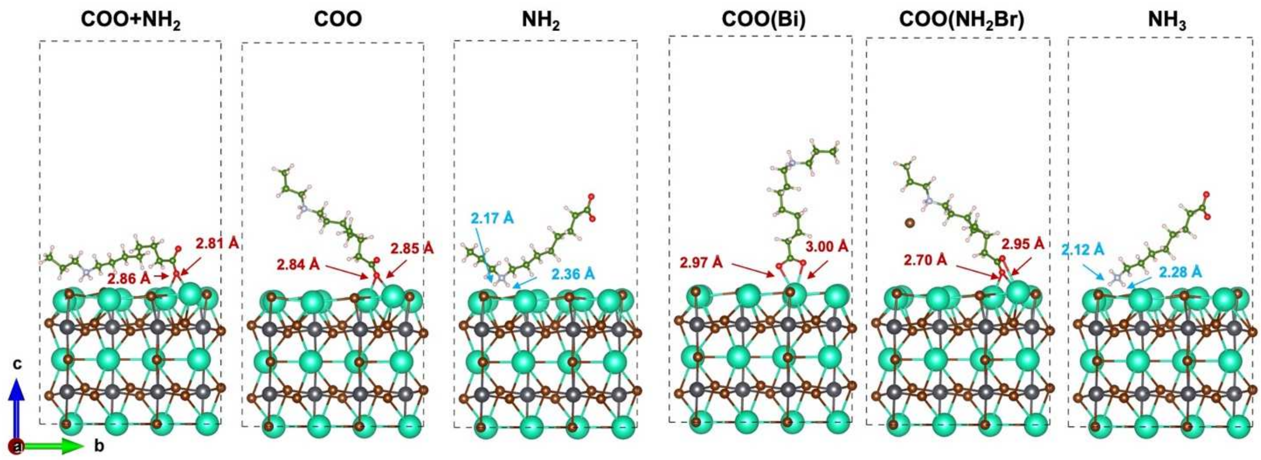

Figure S4. Side view of the optimized zwitterionic molecule adsorbed on $CsPbBr_3$ stoichiometric surface for all anchoring modes considered in this work. O-Cs(surface) and H-Br(surface) distances highlighted in red and blue, respectively. Color legend: C (green), H (light pink), O (red), N (light blue), Cs (turquoise), Pb (grey) and Br (brown).

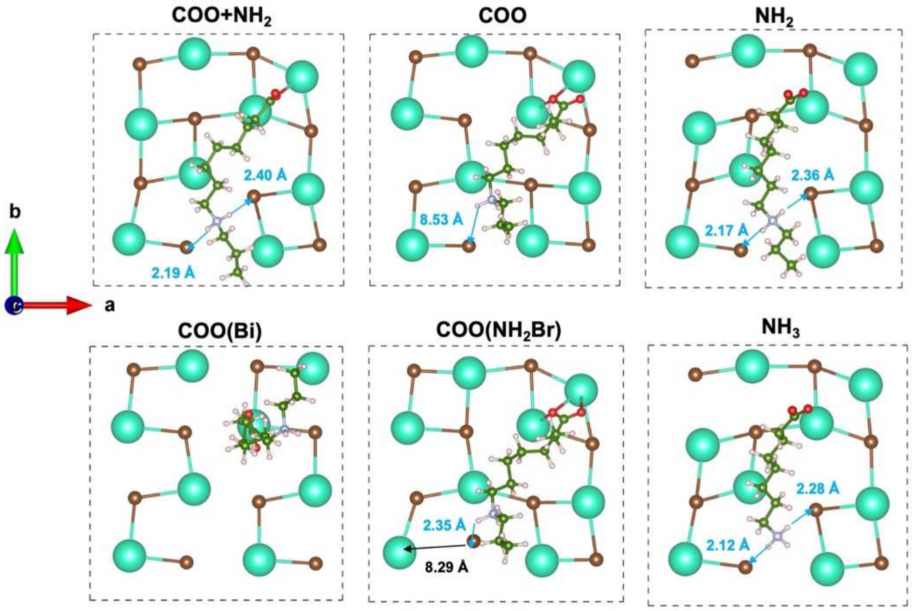

Figure S5. Top view of the optimized zwitterionic molecule adsorbed on $CsPbBr_3$ perovskite stoichiometric surface for all anchoring modes considered in this work. H-Br(surface) highlighted in blue. For the *COO(NH₂Br)* configuration, Br(detached)-Cs(surface) distance is indicated in black. Color legend: C (green), H (light pink), O (red), N (light blue), Cs (turquoise), Pb (grey) and Br (brown).

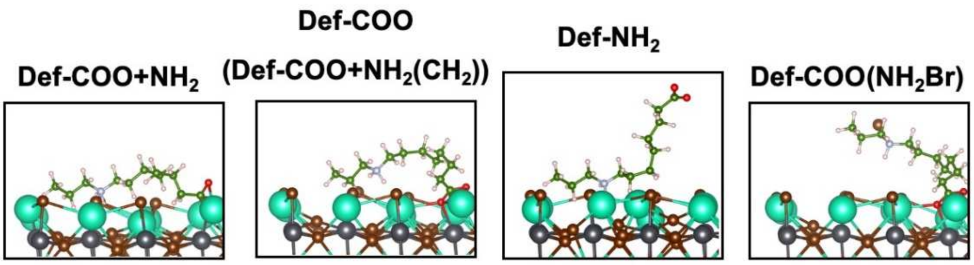

$$E_b = E_{Surf\text{-}Mol} - E_{Surf} - E_{Mol}$$

E_b (eV)	Def-COO+NH_2	Def-COO (Def-COO+NH_2(CH_2))	Def-NH_2	Def-COO(NH_2Br)
High Θ 2x2 Supercell, Vacuum	-5.90	-5.50	Unstable	-3.74
Low Θ 4x4 Supercell, Vacuum	-5.96	-5.77	-5.86	-1.19
Low Θ Cluster, Vacuum	-6.11	-3.98	-6.17	-1.12
Low Θ Cluster, Hexane	-4.44	-2.31	-5.67	-1.61
Low Θ Cluster, DCM	-5.37	-3.23	-5.03	-1.02

Figure S6. Computed binding energies (E_b) for all configurations of the zwitterionic molecule considered in this work on $CsPbBr_3$ perovskite defective surface at different coverages (Θ) and dielectric media. Data highlighted in yellow are featured in Figure 4 of the main text. Color legend: C (green), H (light pink), O (red), N (light blue), Cs (turquoise), Pb (grey) and Br (brown).

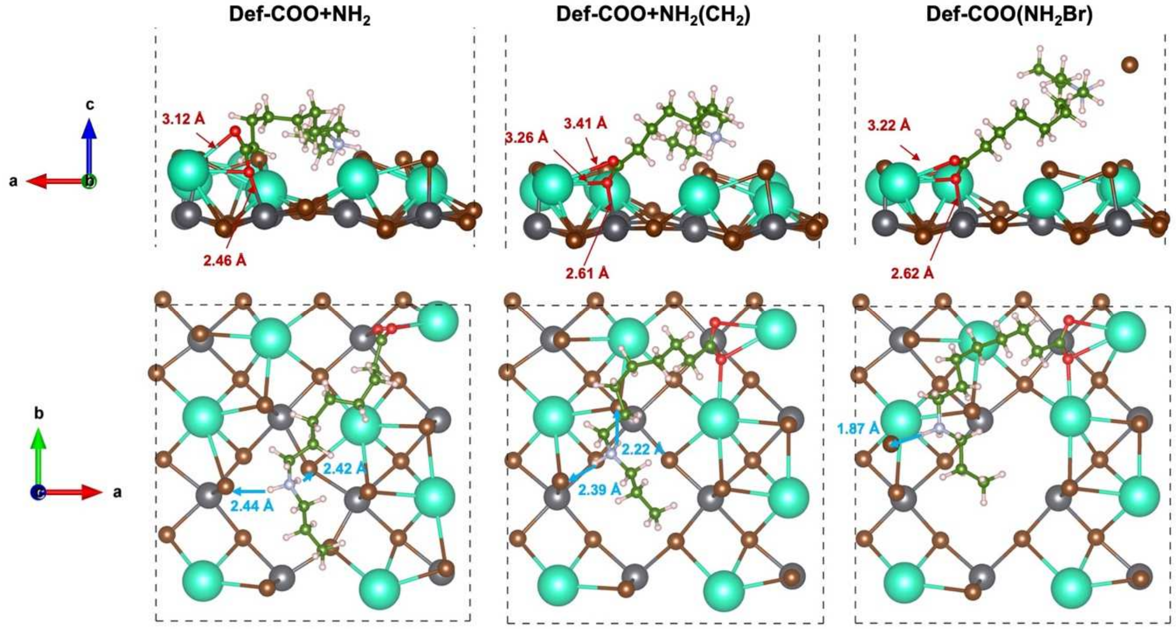

Figure S7. Side and top views of the optimized zwitterionic molecule adsorbed on $CsPbBr_3$ perovskite defective surface for all anchoring modes considered in this work. O-Cs(surface) and H-Br(surface) distances highlighted in red and blue, respectively. Color legend: C (green), H (light pink), O (red), N (light blue), Cs (turquoise), Pb (grey) and Br (brown).

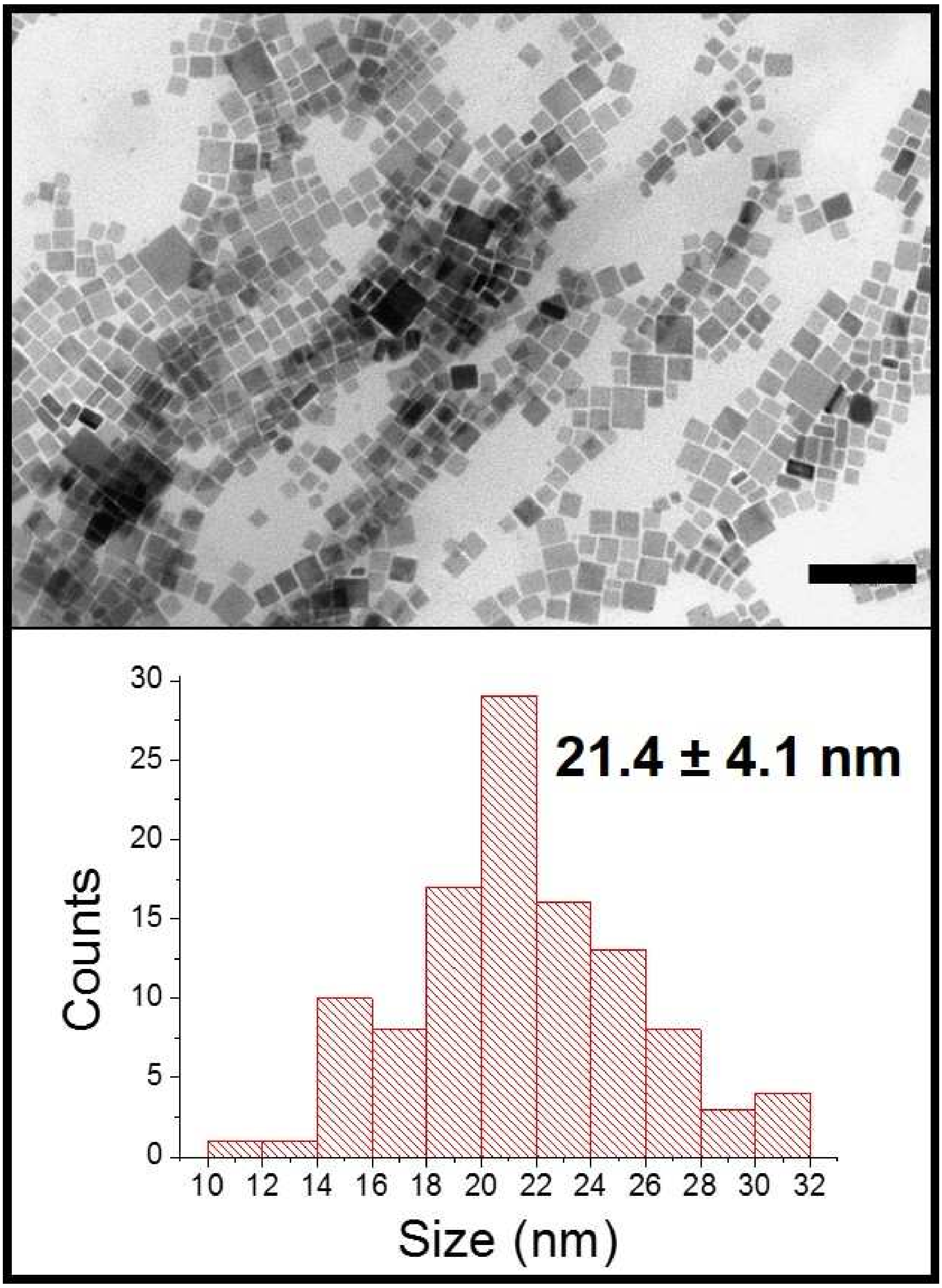

Figure S8. TEM image of $CsPbBr_3$ NCs (scale bar = 100 nm) and the relevant histogram of the size distribution.

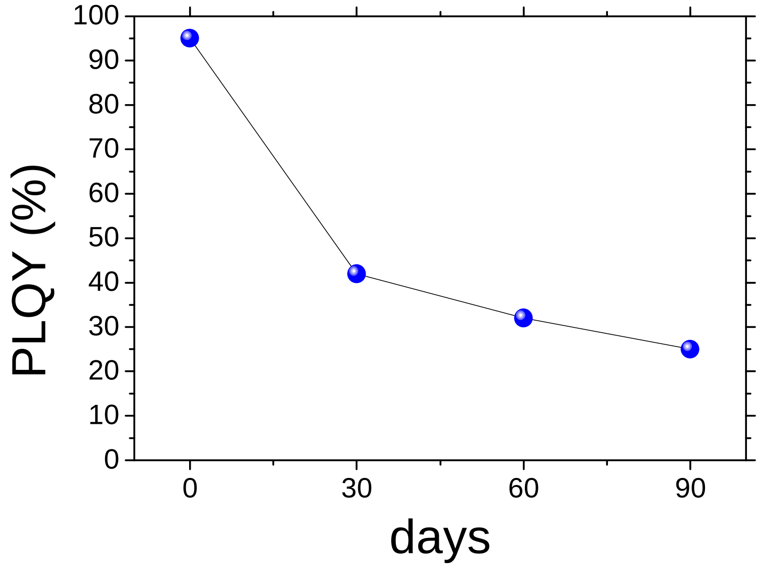

Figure S9. Evolution of the PLQY values of conventional $CsPbBr_3$ NCs in DCM over time.

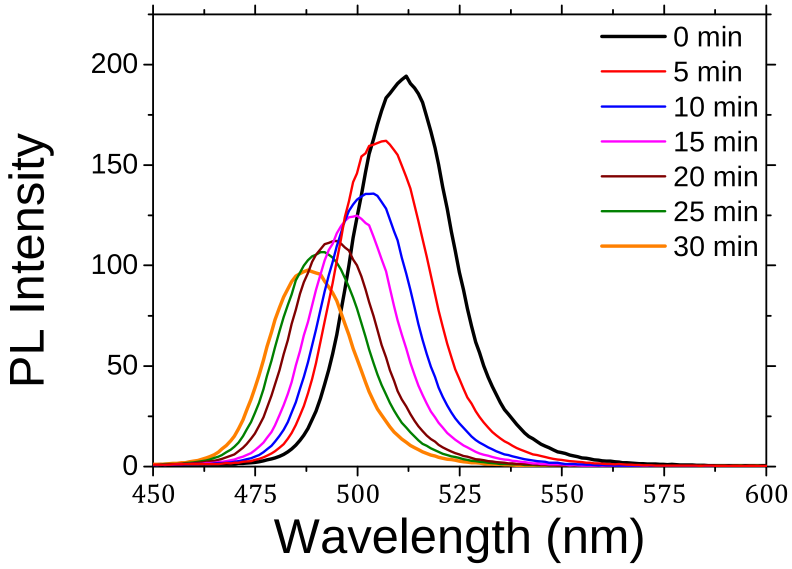

Figure S10. Evolution of the PL spectra of conventional $CsPbBr_3$ NCs under UV irradiation (365 nm, 8 W) in DCM over time.

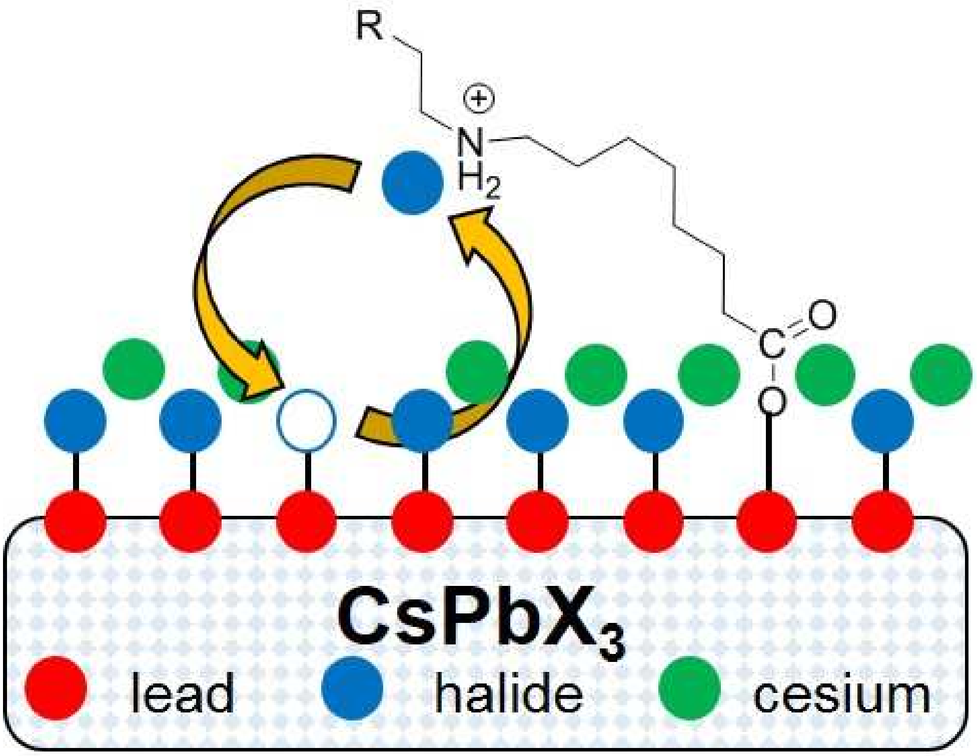

Figure S11. Schematic representation of the equilibria at the organic/inorganic interface of the NC involving the bidentate ligand during the Br→Cl halide exchange. This result is supported by theoretical calculations showing that the interaction of the dialkylammonium group belonging to the zwitterionic ligand with surface bromides could facilitate the halide availability in proximity of the surface during the exchange process. In fact, the mono-anchored configuration, in which the bromide is attached to the dialkylammonium group upon the creation of a surface bromide vacancy (*COO(NH_2Br)* and *Def-COO(NH_2Br)* in Figures S1 and S4, respectively), was found to be stable enough to infer that possible halide voids at the surface can be filled with the ligand relaxation to the bidentate configuration.

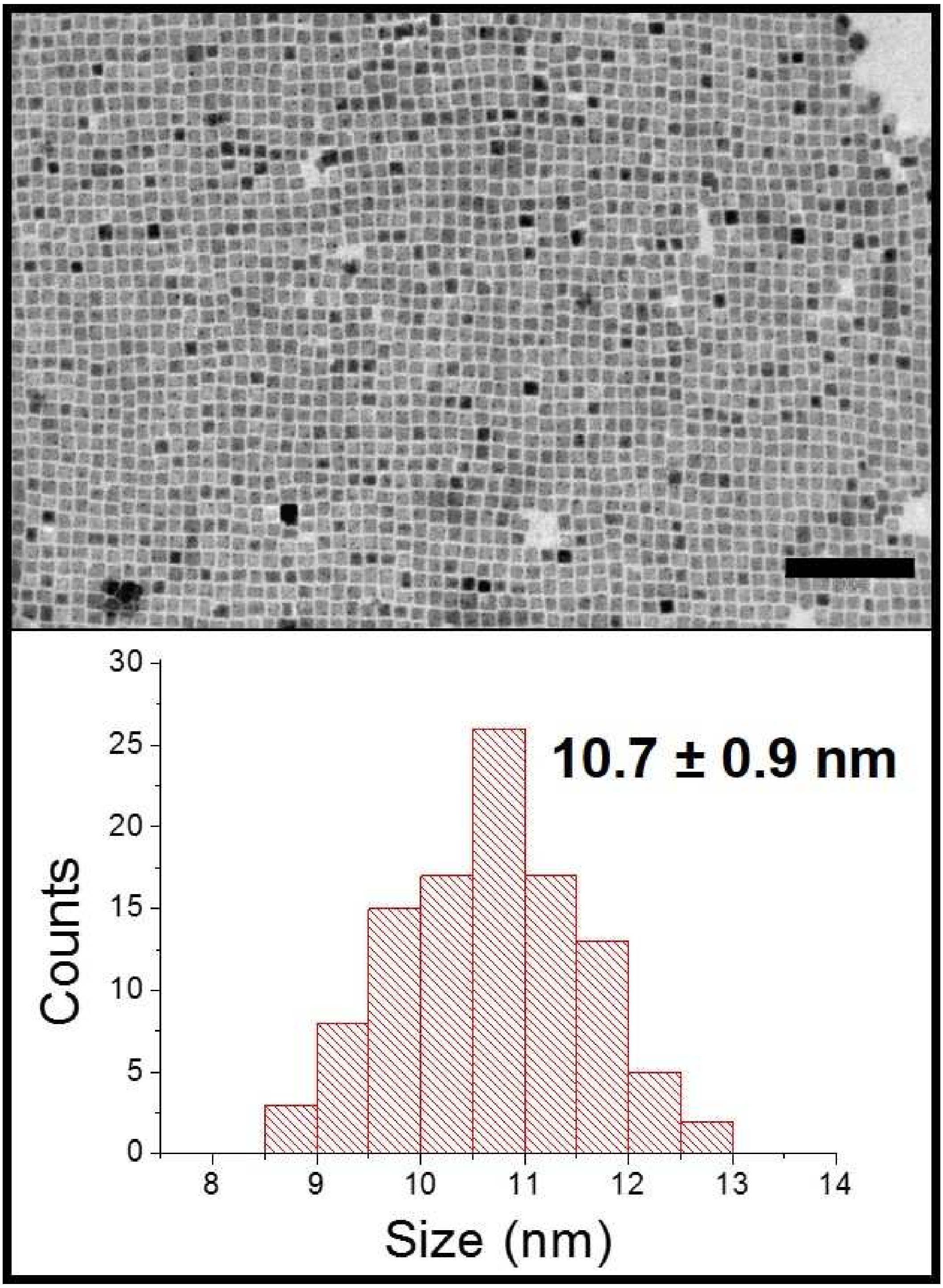

Figure S12. TEM image (scale bar = 100 nm) of $CsPbBr_xI_{3-x}$ NCs (prepared with PbI_2 as the lead precursor in the same reaction conditions described in Figure 1 of the manuscript) and the relevant histogram of the size distribution.

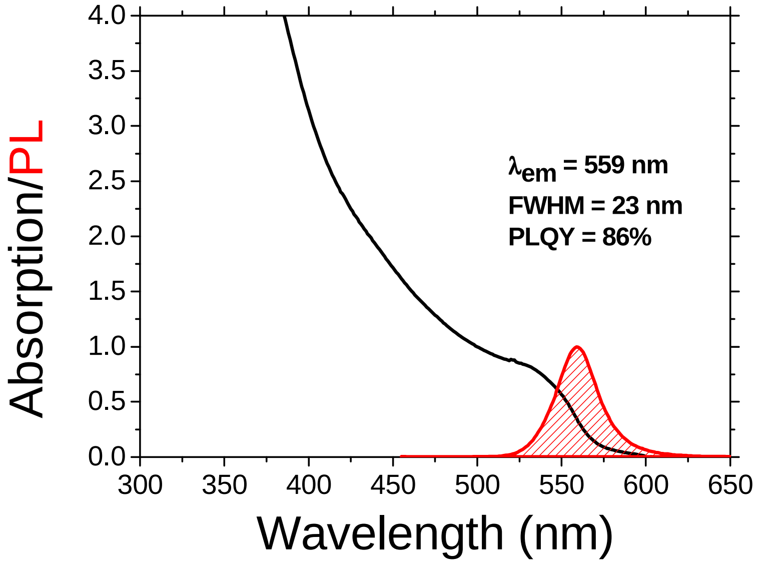

Figure S13. UV-vis and PL spectra of $CsPbBr_xI_{3-x}$ NCs recorded in DCM.

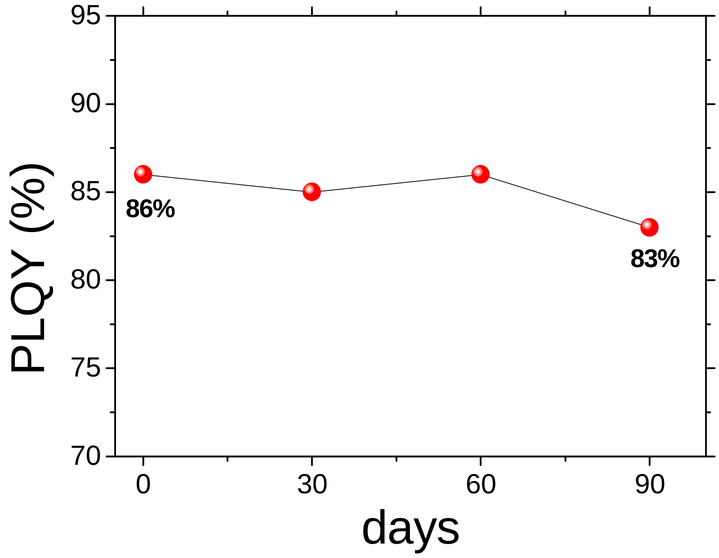

Figure S14. Evolution of the PLQY of the purified $CsPbBr_xI_{3-x}$ NCs stored in DCM under ambient conditions.

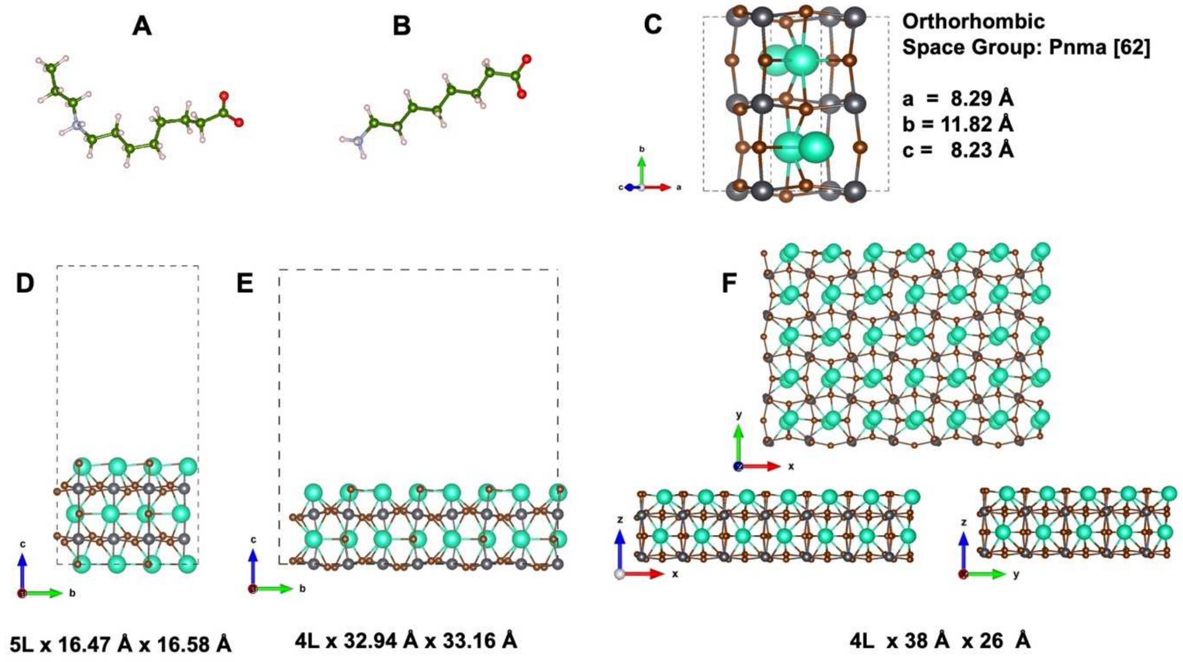

Figure S15. Structural models used in this work. A) Zwitterionic molecule B) primary amine C) Orthorhombic $CsPbBr_3$ unit cell D) 5L 2x2 supercell slab of the $CsPbBr_3$ (001) surface, used for high Θ periodic calculations in vacuum E) 4L 4x4 supercell slab of the $CsPbBr_3$ (001) surface, used for low Θ periodic calculations in vacuum F) 4L cluster of the $CsPbBr_3$ (001) surface used for low Θ non-periodic calculations in vacuum, hexane and DCM. Color legend: C (green), H (light pink), O (red), N (light blue), Cs (turquoise), Pb (grey) and Br (brown).

References

[1] Grabolle, M.; Spieles, M.; Lesnyak, V.; Gaponik, N.; Eychmüller, A.; Resch-Genger, U. Determination of the Fluorescence Quantum Yield of Quantum Dots: Suitable Procedures and Achievable Uncertainties. *Anal. Chem.* **2009**, *81*, 6285–6294.

[2] Burke, K. Perspective on density functional theory. *J. Chem. Phys.* **2012**, *136*, 150901.

[3] Perdew, J. P.; Burke, K.; Ernzerhof, M. Generalized gradient approximation made simple. *Phys. Rev. Lett.* **1996**, *77*, 3865–3868.

[4] Tkatchenko, A.; Scheffler, M. Accurate molecular van der Waals interactions from ground-state electron density and free-atom reference data. *Phys. Rev. Lett.* **2009**, *102*, 073005.

[5] Tkatchenko, A.; DiStasio, R. A.; Car, R.; Scheffler, M. Accurate and efficient method for many-body van der Waals interactions. *Phys. Rev. Lett.* **2012**, *108*, 236402.

[6] Havu, V.; Blum, V.; Havu, P.; Scheffler, M. Efficient O(N) Integration For All-Electron Electronic Structure Calculation Using Numeric Basis Functions. *J. Comp. Phys.* **2009**, *228*, 8367–8379.

[7] Blum,V.; Gehrke, R.; Hanke, F.; Havu, P.; Havu, V.; Ren, X.; Reuter, K.; Scheffler, M. Ab initio molecular simulations with numeric atom-centered orbitals. *Comput. Phys. Commun.* **2009**, *180*, 2175–2196.

[8] Van Lenthe, E.; Snijders, J. G.; Baerends, E. J. The zero-order regular approximation for relativistic effects: the effect of spin–orbit coupling in closed Shell molecules. *J. Chem. Phys.* **1996**, *105*, 6505–6516.

[9] Sinstein, M.; Scheurer, C.; Matera, S.; Blum, V.; Reuter, K.; Oberhofer, H. Efficient Implicit Solvation Method for Full Potential DFT. *J. Chem. Theory Comput.* **2017**, *13*, 5582–5603.

[10] Sun, W.; Ceder, G. Efficient creation and convergence of surface slabs. *Surf. Sci.* **2013**, *617*, 53–59.
